# Trombose de Prótese Mecânica Aórtica em Mulher de 65 Anos com Infecção pelo SARS-CoV-2

**DOI:** 10.36660/abc.20200754

**Published:** 2020-12-01

**Authors:** Marcos H. F. Jacob, Tatiana C.A.T. Leal, Paulo Rogério Soares, Alexandre de Matos Soeiro

**Affiliations:** 1 Instituto do Coração Faculdade de Medicina Universidade de São Paulo São PauloSP Brasil Instituto do Coração (InCor) - Faculdade de Medicina da Universidade de São Paulo,São Paulo, SP – Brasil

**Keywords:** Próteses Valvulares Cardíacas, Trombose, COVID-19, SARS-CoV-2, Fibrilação Atrial, Diagnóstico por Imagem, Infarto do Miocárdio

## Dados clínicos

Paciente feminina de 65 anos internada inicialmente devido a um episódio de melena, sendo suspensa a anticoagulação e realizada endoscopia digestiva alta que não detectou sangramentos. Tinha antecedentes de pós-operatório tardio em 2005 de prótese mecânica aórtica e cirurgia de Bentall – de Bono devido a dissecção de aorta Stanford A. Era ex-tabagista, com obesidade e fibrilação atrial crônica em uso regular de varfarina. No dia seguinte, apresentou infarto agudo do miocárdio com supradesnivelamento de segmento ST em parede anterior, sendo submetida à angioplastia com *stent* farmacológico de descendente anterior e recebendo aspirina, clopidogrel 75 mg ao dia e anticoagulação com enoxaparina 100 mg 12/12 h. Na ocasião, realizou ecocardiograma transtorácico que demonstrou disfunção ventricular leve (FEVE: 50%) e folhetos de prótese aórtica com mobilidade preservada; o gradiente sistólico ventrículo esquerdo-aorta máximo era de 58 mmHg e o médio era de 33 mmHg. Dois dias após, iniciou quadro de anosmia, cefaléia, pico subfebril e hipoxemia, sendo confirmado diagnóstico de SARS-CoV-2 por PCR em swab de orofaringe. Passados sete dias, ainda em uso de enoxaparina plena, a paciente iniciou quadro de dor torácica atípica associado à dispneia. Ao exame físico apresentava-se em regular estado geral, dispneica, acianótica, pulsos presentes e cheios nos 4 membros, Glasgow de 15, pressão arterial de 120 x 86 mmHg, frequência cardíaca de 130 bpm, oximetria periférica de 97% em uso de cateter nasal de oxigênio a 3 L/min e tempo de enchimento capilar menor que 3 segundos. Ictus cordis na linha hemiclavicular esquerda, a nível do 5º espaço intercostal. Bulhas cardíacas normofonéticas, com sopro sistólico ejetivo de moderada intensidade com click metálico em foco aórtico presente. Fígado não palpável e estertores crepitantes discretos e esparsos em campos pulmonares bilateralmente.

### Exames complementares

**Eletrocardiograma:** Taquicardia sinusal com supradesnível de ST em parede anterior.

**Figura 1:**
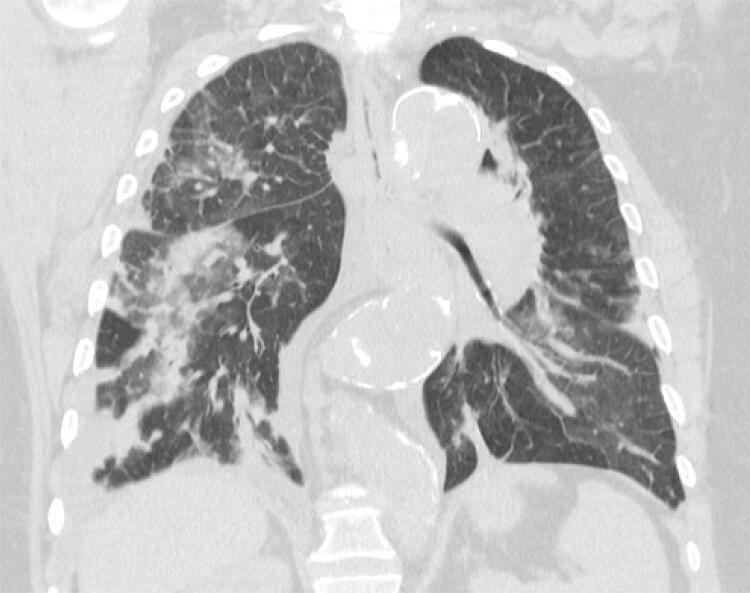
Imagem tomográfica em plano coronal mostrando múltiplas opacidades pulmonares em vidro fosco características da infecção pela COVID-19.

**Tomografia de tórax:** Múltiplas opacidades pulmonares em vidro fosco, parte delas associadas à espessamento septal de permeio e outras confluindo em pequenos focos de consolidação, com distribuição multifocal bilateral e predominantemente periférica, nos campos médios e inferiores ( Figura 1 ).

**Figura 2:**
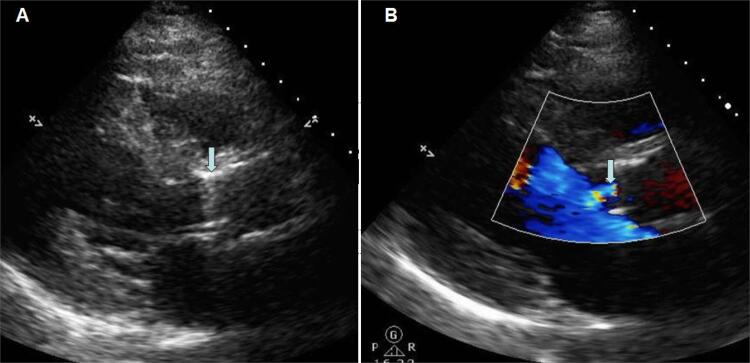
Janela paraesternal longitudinal do ecocardiograma transtorácico evidenciando abertura mínima da prótese valvar aórtica (seta à esquerda) durante a sístole (A) e no modo Doppler colorido (B), bem como a presença de fluxo laminar através da prótese (seta à direita). Achados que sugerem aumento do gradiente transvalvar e trombose aguda de prótese.

**Ecocardiograma:** Ventrículo esquerdo com função sistólica diminuída às custas de acinesia do septo e do segmento apical da parede inferior com fração de ejeção estimada em 40%. Prótese mecânica com difícil caracterização da mobilidade de seus elementos, aparentemente com redução de mobilidade de um desses. Observada discreta regurgitação ao Doppler e mapeamento de fluxo em cores. Gradiente sistólico ventrículo esquerdo-aorta estimado máximo de 79 mmHg e médio de 48 mmHg (aumento de 15 mmHg em relação ao exame de dez dias anteriores). A velocidade máxima na prótese aórtica foi estimada em 4,47 m/s. A relação das velocidades entre a via de saída do ventrículo esquerdo e da prótese aórtica foi estimada em 0,21. O tempo de aceleração na prótese valvar aórtica foi estimado em 105 ms. A via de saída do ventrículo esquerdo foi estimada em 2,2 cm ( Figura 2 ).

**Figura 3:**
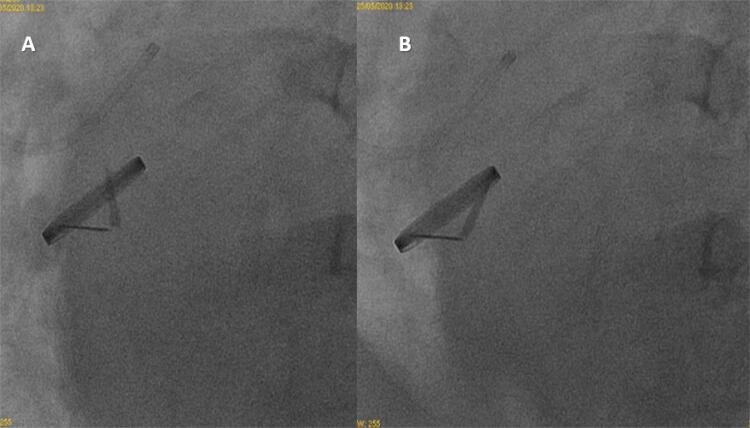
Cineangiocoronariografia demonstrando redução importante da mobilidade do folheto da prótese aórtica em sístole (A) e diástole (B).

**Cineangiocoronariografia:** Não evidenciou nova lesão coronariana, mas demonstrou redução importante de mobilidade de um dos folhetos da prótese aórtica ( Figura 3 ).

**Diagnóstico clínico:** Trombose aguda de prótese mecânica aórtica em paciente com infecção pelo Sars-CoV-2 e infarto agudo do miocárdio recente.

**Raciocínio clínico:** A paciente em questão havia sido submetida a intervenção coronariana recente de descendente anterior devido a infarto agudo do miocárdio com supradesnivelamento de segmento ST, sendo aventada a hipótese inicial de trombose aguda de *stent* , portanto a paciente foi prontamente submetida a novo cateterismo. Devido à redução da mobilidade da prótese identificada no exame, à ausência de novas lesões coronarianas, associadas com os achados clínicos de sopro de estenose aórtica e ecocardiográficos de aumento do gradiente sistólico médio na valva aórtica, suspeitou-se de trombose aguda de prótese valvar. Devido a um episódio de sangramento recente, a trombólise química foi contraindicada, sendo procedida à cirurgia. No intraoperatório, foi evidenciada prótese mecânica de aspecto anatômico normal, porém com um dos folhetos fixo por trombo ao redor, confirmando o diagnóstico. Foi realizada trombectomia mecânica e limpeza de ambos os folhetos, com normalização da mobilidade.

### Evolução clínica

Manteve-se estável imediatamente após a cirurgia, porém evoluiu em insuficiência respiratória pelo SARS-CoV-2, progrediu com hipoxemia grave e choque séptico refratário, evoluindo a óbito.

## Comentários

A infecção pelo SARS-CoV-2 gera alterações hemostáticas como alargamento do INR, plaquetopenia e aumento de produtos de degradação da fibrina que estão relacionados a trombogênese.^1^ Essas alterações podem ser decorrentes de um efeito específico do vírus ou consequência da cascata inflamatória de citocinas que precipita o aparecimento de síndrome da resposta inflamatória sistêmica,^2^ como observado em outras doenças virais. Estudos de necrópsia demonstraram presença de microtrombos em alvéolos pulmonares, diferenciando a infecção pelo SARS-CoV-2 de outras infecções virais.^3^ Dentre as manifestações clínicas da trombogênese relacionada à infecção pelo SARS-CoV-2 podemos destacar a síndrome coronariana aguda, coagulação intravascular disseminada, acidente vascular encefálico, tromboembolismo venoso ou arterial sendo a trombose aguda de prótese valvar ainda não descrita.^4^

A taxa de incidência anual de trombose de prótese valvar varia de 0,1% a 5,7% sendo mais comum em próteses mecânicas, no período pós-operatório precoce, em posições mitral e tricúspide e em casos de anticoagulação subterapêutica.^5^ Nesse caso, a hipercoagulabilidade representada pela infecção pelo SARS-CoV-2 em conjunto com as alterações hemodinâmicas causadas pelo infarto agudo do miocárdio recente, que gera um miocárdio atordoado e consequente redução da diluição e *washout* dos fatores ativadores da coagulação periprótese, contribuíram para o mecanismo trombótico. Mesmo em uso de anticoagulação plena subcutânea, a paciente apresentou trombose da prótese valvar provavelmente relacionada à infecção viral, demonstrando mais uma das formas variadas de manifestações pró-coagulantes do SARS-CoV-2.^5^
