# Supplementary material for: Trombose de Prótese Mecânica Aórtica em Mulher de 65 Anos com Infecção pelo SARS-CoV-2
Source: Arq Bras Cardiol. 2020 Dec 1;115(6):1180–3. [Article in Portuguese] doi: 10.36660/abc.20200754 (PMC8133743; doi:10.36660/abc.20200754)
Supplement: Translation

# Translation: Mechanical Aortic Prosthetic Thrombosis in a 65-Year-Old Woman with SARS-CoV-2 Infection

**Authors:** Marcos H. F. Jacob, Tatiana C.A.T. Leal, Paulo Rogério Soares, Alexandre de Matos Soeiro

**Affiliations:** 1 Brazil Instituto do Coração (InCor) - Faculdade de Medicina da Universidade de São Paulo, São Paulo, SP – Brazil

**Keywords:** Heart Valve Prosthesis, Thrombosis, COVID-19, SARS-CoV-2, Atrial Fibrillation, Atrial Fibrillation, Diagnosis, Imaging, Myocardial Infarction

## Clinical data

A 65-year-old female patient was hospitalized, initially due to an episode of melena. Anticoagulation was suspended, and upper gastrointestinal endoscopy was performed, without detecting bleeding. In 2005, she had late post-operative history of mechanical aortic prosthesis and Bentall and de Bono surgery due to Stanford A aortic dissection; she was a former tobacco user, with obesity and chronic atrial fibrillation under regular use of warfarin. The following day, she had an acute myocardial infarction with ST-segment elevation in the anterior wall; she was submitted to angioplasty with an anterior descending drug-eluting stent, and she received aspirin, clopidogrel 75 mg daily, and anticoagulation with enoxaparin 100 mg every 12 hours. On that occasion, she underwent transthoracic echocardiogram that demonstrated mild ventricular dysfunction (LVEF: 50%) and aortic prosthesis leaflets with preserved mobility; the peak systolic pressure gradient between the left ventricle and the aorta was 58 mmHg, with a mean of 33 mmHg. Two days later, she began to present anosmia, headache, subfebrile temperature, and hypoxemia, and diagnosis of SARS-CoV-2 was confirmed by oropharyngeal swab PCR. Seven days later, while still on full enoxaparin, the patient started presenting atypical chest pain associated with dyspnea. During physical examination, the patient’s overall condition was regular; she had dyspnea, without cyanosis. Pulse was present and full in all 4 limbs, and she had Glasgow score of 15, blood pressure 120/86 mmHg, heart rate 130 bpm, peripheral oximetry 97% while using a nasal oxygen catheter at 3 L/min, and capillary filling time of less than 3 seconds. Ictus cordis was in the left hemiclavicular line, at the level of the fifth intercostal space. She had normophonetic heart sounds, with moderate intensity ejective systolic murmur with metallic click in aortic focus. The patient’s liver was not palpable, and there were mild and sparking crackles in the pulmonary fields bilaterally.

### Complementary exams

**Electrocardiogram:** Sinus tachycardia with ST elevation in the anterior wall.

**Chest tomography:** Multiple ground-glass pulmonary opacities, some associated with intervening septal thickening and others converging in small foci of consolidation, with bilateral, multifocal, and predominantly peripheral distribution, in the middle and lower fields ( Figure 1 ).

**Figure 1:** Coronal plane tomography image showing multiple ground-glass pulmonary opacities characteristic of COVID-19 infection.

**Echocardiogram:** The left ventricle showed decreased systolic function due to akinesia of the septum and the apical segment of the lower wall with an estimated ejection fraction of 40%. It was difficult to characterize of the mobility of the elements of the mechanical prosthesis; one of which apparently had reduced mobility. Discreet regurgitation was observed on Doppler and color flow mapping. The peak systolic pressure gradient between the left ventricle and the aorta was estimated at 79 mmHg, with a mean of 48 mmHg (increase of 15 mmHg compared to the exam 10 days prior). The maximum velocity of the aortic prosthesis was estimated at 4.47 m/s. The ratio of velocities of the left ventricular outflow tract and the aortic prosthesis was estimated at 0.21. The acceleration time in the aortic valve prosthesis was estimated at 105 ms. The left ventricular outflow tract was estimated at 2.2 cm ( Figure 2 ).

**Figure 2:** Longitudinal parasternal window of transthoracic echocardiogram showing minimal opening of the aortic valve prosthesis (left arrow) during systole (A) and in color Doppler mode (B), as well as the presence of laminar flow through the prosthesis (right arrow). These findings suggest an increase in the transvalvular gradient and acute prosthesis thrombosis.

**Coronary cineangiography:** It showed no evidence of new coronary lesion, but it did demonstrate an important decrease in the mobility of one of the aortic prosthesis leaflets ( Figure 3 ).

**Figure 3:** Coronary cineangiography demonstrating important decrease in the mobility of the aortic prosthesis leaflet during systole (A) and diastole (B).

**Clinical diagnosis:** Acute thrombosis of mechanical aortic prosthesis in a patient with Sars-CoV-2 infection and recent acute myocardial infarction.

**Clinical reasoning:** The patient in question had recently undergone anterior descending coronary intervention due to acute myocardial infarction with ST-segment elevation, and the initial hypothesis of acute stent thrombosis was raised; the patient was, therefore, promptly submitted to a new catheterization. Due to the reduced mobility of the prosthesis identified in the exam and to the absence of new coronary lesions, associated with the clinical findings of aortic stenosis murmur and echocardiographic findings of increased mean systolic gradient in the aortic valve, acute valve prosthetic thrombosis was suspected. Due to a recent bleeding episode, chemical thrombolysis was contraindicated, and surgery was performed. During the intraoperative period, the mechanical prosthesis had a normal anatomical aspect, but one of the leaflets was fixed by a surrounding thrombus, thus confirming the diagnosis. Mechanical thrombectomy was performed with cleaning of both leaflets, and mobility returned to normal.

### Clinical evolution

The patient remained stable immediately after surgery, but she evolved with respiratory failure due to SARS-CoV-2, progressing with severe hypoxemia and refractory septic shock, progressing to death.

## Comments

SARS-CoV-2 infection causes hemostatic changes such as enlargement of the INR, thrombocytopenia, and increased products of fibrin degradation that are related to thrombogenesis.^1^These changes may be due to a specific effect of the virus or a consequence of the inflammatory cytokine cascade that precipitates the onset of systemic inflammatory response syndrome,^2^as observed in other viral diseases. Necropsy studies have demonstrated the presence of microthrombi in pulmonary alveoli, differentiating SARS-CoV-2 infection from other viral infections.^3^Among the clinical manifestations of thrombogenesis related to SARS-CoV-2 infection, we may highlight acute coronary syndrome, disseminated intravascular coagulation, stroke, and venous or arterial thromboembolism; acute thrombosis of a prosthetic valve has not yet been described.^4^

The annual incidence rate of valve prosthetic thrombosis varies from 0.1% to 5.7%, and it is more common in mechanical prostheses, during the early postoperative period, in mitral and tricuspid positions, and in cases of subtherapeutic anticoagulation.^5^In this case, the hypercoagulability represented by SARS-CoV-2 infection, in conjunction with the hemodynamic changes caused by the recent acute myocardial infarction, which generates a stunned myocardium with consequent reduction in dilution and washout of the periprosthetic coagulation activating factors, contributed to the thrombosis of the mechanism. Even under full subcutaneous anticoagulation, the patient presented thrombosis of the valve prosthesis, which was probably related to viral infection, demonstrating yet another of the various forms of pro-coagulant manifestations of forms of SARS-CoV-2.^5^
